# CaMuS: simultaneous fitting and de novo imputation of cancer mutational signature

**DOI:** 10.1038/s41598-020-75753-8

**Published:** 2020-11-09

**Authors:** Maria Cartolano, Nima Abedpour, Viktor Achter, Tsun-Po Yang, Sandra Ackermann, Matthias Fischer, Martin Peifer

**Affiliations:** 1 Department of Translational Genomics, Center of Integrated Oncology Cologne-Bonn, Medical Faculty, University of Cologne, Robert-Koch-Str. 21, 50931 Cologne, Germany; 2 Center for Molecular Medicine Cologne, Medical Faculty, University Hospital Cologne, Cologne, Germany; 3 Computing Center, University of Cologne, Cologne, Germany; 4 Department of Experimental Pediatric Oncology, University Children’s Hospital of Cologne, Medical Faculty, Cologne, Germany

**Keywords:** Paediatric cancer, Cancer genomics

## Abstract

The identification of the mutational processes operating in tumour cells has implications for cancer diagnosis and therapy. These processes leave mutational patterns on the cancer genomes, which are referred to as mutational signatures. Recently, 81 mutational signatures have been inferred using computational algorithms on sequencing data of 23,879 samples. However, these published signatures may not always offer a comprehensive view on the biological processes underlying tumour types that are not included or underrepresented in the reference studies. To circumvent this problem, we designed *CaMuS* (Cancer Mutational Signatures) to construct de novo signatures while simultaneously fitting publicly available mutational signatures. Furthermore, we propose to estimate signature similarity by comparing probability distributions using the Hellinger distance. We applied *CaMuS* to infer signatures of mutational processes in poorly studied cancer types. We used whole genome sequencing data of 56 neuroblastoma, thus providing evidence for the versatility of *CaMuS*. Using simulated data, we compared the performance of *CaMuS* to *sigfit*, a recently developed algorithm with comparable inference functionalities. *CaMuS* and *sigfit* reconstructed the simulated datasets with similar accuracy; however two main features may argue for *CaMuS* over *sigfit*: (i) superior computational performance and (ii) a reliable parameter selection method to avoid spurious signatures.

## Introduction

A cell encounters several sources of DNA damage throughout its life cycle that can result into a mutation. The exposure to exogenous mutagens is a renowned cause of DNA damage. Similarly, endogenous processes intrinsic to DNA replication, chemical modification, and repair can cause DNA damage when not correctly functioning^1^. Often these mutational processes leave a distinct fingerprint on the DNA of the cancer cells. A classic example is the enrichment in C>T nucleotide transitions induced by UV light exposure in skin cancers^2^. Likewise, the genome of lung cancer patients with a history of tobacco smoking shows C>A transversions induced by carcinogens exposure^3^. Generally, the straightforward identification of the mutation patterns in cancer cells (henceforth called mutational signatures) can be hampered by the presence of multiple mutational processes each contributing its own spectrum of somatic mutations. To address this problem, nonnegative matrix factorization (NMF) has been initially applied to deconvolute somatic mutations in to mutational signatures^4,5^. Within this framework, single nucleotide variants (SNVs) are classified based on their type (C>A, C>G, C>T, T>A, T>C, T>G, when strand symmetry is assumed) and their genomic context (5′ and 3′ neighbour of the mutated nucleotide). Upon NMF, such data is clustered into non-negative probability distributions (i.e., mutational signatures) and their contribution to each sample analysed. This concept has been recently extended to doublet base substitutions (DBSs), and small insertions and deletions (IDs)^6^. To date, a reference catalogue of 81 SNVs, DBSs, and IDs mutational signatures extracted across multiple human cancer types is available, including the presumable aetiology for a subset of them^6^. Since several mutational processes may have clinical implications, scientists have proposed additional computational methods to improve the identification of the mutational signatures^7^. These approaches can be broadly classified into two main types: (i) methods to infer mutational signatures, and (ii) algorithms aimed at linearly fitting reference signatures to mutation data^5,6,8–11^. While unsupervised clustering provided by methods of type (i) has the advantage of describing potentially novel mutational signatures, it may introduce biases during signature estimation if the sample size is small. In this setting, it may be preferable to determine the contribution of the reference signatures in each sample by linear fitting.

In order to combine both approaches we have developed *CaMuS*. Our algorithm initially fits reference signatures to mutation data (i.e., SNVs, DBSs and IDs) and subsequently performs NMF on the unexplained data to infer potentially novel patterns of mutational processes. Furthermore, *CaMuS* measures signature similarity by the Hellinger distance, which is specifically designed to compare probability distributions. To our knowledge, there is only one other method, called *sigfit*, combining signature fitting and extraction in a single inferential process^12^. Recently, *sigfit* has been successfully applied to describe the mutational processes underlying the evolution of canine transmissible venereal tumour^13^. By using synthetic data, we demonstrate that *CaMuS* is able to reconstruct the simulated data with a superior computational performance. Furthermore, *sigfit* does not offer a straightforward functionality for parameter selection, which may potentially lead to signature overfitting. Finally, we applied *CaMuS* to treatment naive neuroblastoma (NB) genomes to show its versatility in a setting of small sample sizes of poorly characterised tumours.

## Methods

### Implementation

**Figure 1:**
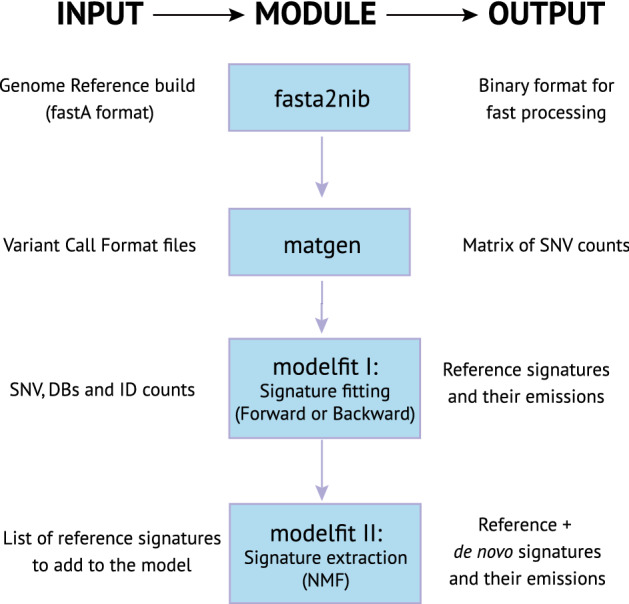
Schematic representation of *CaMuS*’ workflow. *CaMuS* is a command line based tool consisting of 3 modules: *fasta2nib*, *matgen,* and *modelfit*. *fasta2nib* converts any reference genome in to binary formatted individual chromosomes. *matgen* uses the chromosomes generated by *fasta2nib* and the variant call files (vcf) to extract the sequence context of each variant and generate a matrix of SNV counts. *modelfit* uses the somatic mutations counts and the reference signatures to perform three types of analyses: (i) NMF-based de novo signature extraction, (ii) linear fitting of the somatic variants to the reference signatures, (iii) de novo signature extraction of the somatic variants left unexplained by linear fitting to reference signatures.

*CaMuS* is implemented in C++ and as a command line tool. An overview of its workflow is presented in Fig. 1. Input data can be provided in the form of variant call format (VCF) files or as a count matrix of the different mutation types. In case of SNVs, such a matrix can be generated directly by *CaMuS*. For IDs and DBSs, the corresponding matrix can be calculated using SigProfilerMatrixGeneratorR^14^. *CaMuS* outputs emissions, signatures as well as Hellinger distance matrices of the selected mutation profiles to reference signatures. *CaMuS* also generates a variety of graphs displaying the signatures of mutational processes as mutational probabilities, stacked bar graphs and hierarchical clustering of the signatures contributions. *CaMuS* source code together with a user manual can be downloaded under: https://www.uni-koeln.de/med-fak/camus/camus.tgz. An example of how to run *CaMuS* is given in the user manual.

### Linear fitting of a reference panel of mutational signatures to the somatic mutations

The matrix of somatic mutation counts *A* (columns are the *n*_*t*_ combinations of mutation types and each of the *n*_*g*_ rows is referring to a sample) is represented as product of two non-negative matrices *W* and *H*: $A \approx W \cdot H$. Here, *H* is the mutational signature matrix and is of dimension $n_{k} \times n_{t}$, where *n*_*k*_ is the number of signatures. In addition, each row of *H* sums to one. The $n_{g} \times n_{k}$ matrix *W* is the so-called emission matrix that reflects the number of mutations associated to each signature of every sample. *CaMuS* initially infers the contribution *W* of the reference signatures *H* from the somatic mutation counts *A*. Thus, as *A* and *H* are provided, only *W* has to be imputed. Reference signatures *H* are fitted to the input data *A* by minimising the sample size normalised Frobenius norm $C = n_{g}^{ - 1} \cdot \parallel A - W \cdot H\parallel_{F}$ (i.e., cost function). This optimisation is performed by solving a non-negative least-squares problem (NNLS) while maintaining the constraint $W_{ij } \ge 0 \;(i = 1, \ldots ,n_{g} \;{\text{and}}\; j = 1, \ldots ,n_{k} )$^15^. In order to prevent overfitting either forward or backward selection of reference signatures is applied. Forward selection starts with an empty model and reference signatures having the strongest impact on the cost function are iteratively added to the model. In contrast, backward feature selection starts with the full model and the reference signatures contributing least to the cost are iteratively discarded. Both forward and backward feature selection is terminated when all signatures have been evaluated. The user is then required to select the set of reference signatures (of size $n_{{k_{ref} }}$) that notably reduces the cost function $C$ (see below in the "Results" section).

### De novo signature inference from data unexplained by the fitting procedure

*CaMuS* reconstructs de novo signature on the data left unexplained by the reference signatures. To this end, the matrix $A^{\prime}$ of residual SNV counts is defined as $A^{\prime} = A - W \cdot H$. Furthermore, the matrix $H^{\prime}$ of dimension $(n_{{k_{ref} }} + n_{{k_{new} }} ) \times n_{t}$ is defined consisting of the fixed $n_{{k_{ref} }}$ reference signatures and $n_{{k_{new} }}$ de novo signatures. The NMF algorithm is thus applied such that $A^{\prime} \approx W^{\prime} \cdot H^{\prime}$. To avoid overfitting, cross-validation is performed: for each $n_{{k_{new} }}$, $n_{g} /k$ models are trained by leaving $k$ samples out and imputing $W^{\prime}_{ - k}$ and $H^{\prime}_{ - k}$, where the index $- k$ denotes that $k$ samples were left-out. Next, contributions $W^{\prime}_{k}$ of the left-out samples is inferred from $A^{\prime} \approx W^{\prime}_{k} \cdot H^{\prime}_{ - k}$, where $A^{\prime}_{k}$ is residuals of the SNV counts of the $k$ left-out samples. Finally, the cost is computed from $A^{\prime}_{k} - W^{\prime}_{k} \cdot H^{\prime}_{ - k}$ and averaged over all samples. Similarly, the cost for the training set is determined from $A^{\prime}_{ - k} - W^{\prime}_{ - k} \cdot H^{\prime}_{ - k}$. Of note, for $n_{{k_{ref} }} = 0$, *CaMuS* only performs de novo signature reconstruction.

### Estimation of signature similarity

To estimate signature similarity between the reference set (e.g., from the COSMIC database) and the inferred de novo signatures, *CaMuS* uses the Hellinger distance. This distance measure is specifically designed to compare probability distributions, which is suited to mutational signatures given that they sum up to one. The Hellinger distance results in values between zero and one. Two mutational profiles are called identical when the Hellinger distance is zero (Fig. S1).

### Data simulation

Simulated data were generated to test *CaMuS* performance. We simulated three instances, where six mutational processes (the Cosmic v2: Signature.1, Signature.3, Signature.5, Signature.11, Signature.18, and Signature.22) were used with varying signature contributions: (i) one dominant mutational process and five low contributing signatures (referred to as Simulation A); (ii) two dominant mutational processes and four low contributing signatures (Simulation B); (iii) all signatures equally contributing (Simulation C). Each instance has been simulated with varying sample size (15, 25, and 50 samples) and number of mutations (500, 1000, 2500, 5000 for signature fitting and 20,000 and 35,000 mutations for de novo signatures inference). In our simulations, we assume that SNVs generated from the mutational processes follow a multinomial probability distribution. Here, success probabilities are chosen to be proportional to the emission of each mutational process. The R script used for generating these simulations and the used data sets are available in the Supplementary Material.

### Analysis of the simulated data with *sigfit*

*Sigfit* was applied to the simulated data using 1000 warmup iterations and 2000 sampling iterations. For de novo inference we used the “*NMF”* method.

### Runtime comparison

To assess the computational performance of *CaMuS* and *sigfit* we measured walltime and memory usage. The analysis was carried out on a single core of an Intel Xeon X5650, 2.67-GHz processor. All results are provided as averages together with their standard deviations calculated from running each setting 50 and 30 times for *CaMuS* and *sigfit*, respectively. The simulated datasets used for this analysis can be found in the Supplementary Materials and the results are showed in Figs. 3, 4, and Table S1.

## Results

### Fitting reference signatures: backward versus forward parameter selection

**Figure 2:**
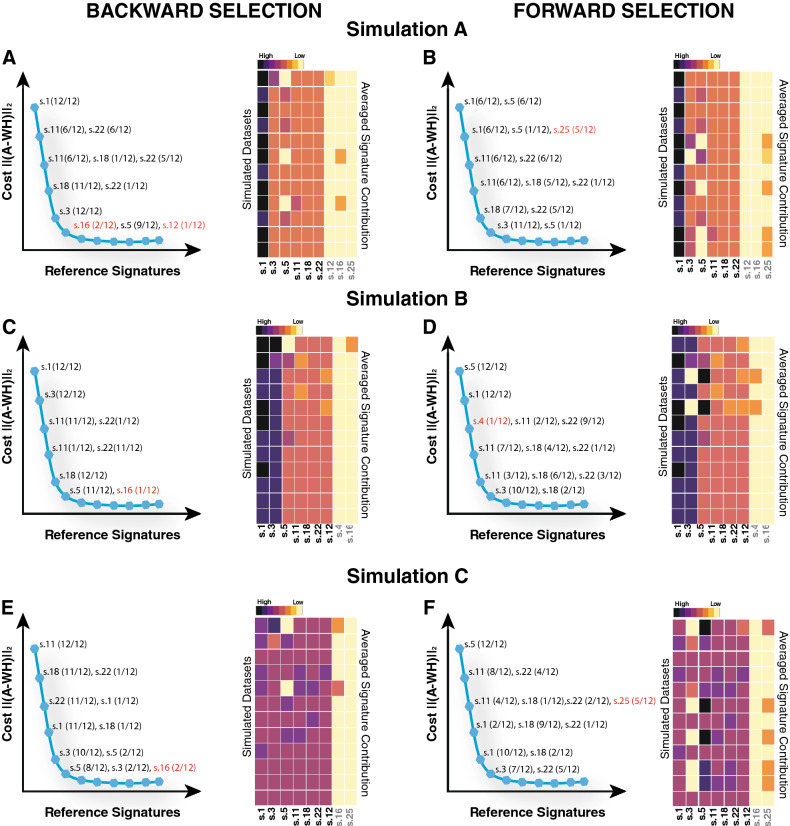
Comparison between backward and forward feature selection. The analysis is shown for three synthetic datasets: Simulation A (top), Simulation B (centre), Simulation C (bottom). Each simulation consists of 12 datasets. Diagram of the cost curve generated by *CaMuS* at the end of the backward (**A**,**C**,**E** left) and of the forward fitting procedure (**B**,**D**,**F** left). At the right of each data point of the cost curve, the signatures and the frequency at which they are scored across all simulated datasets is indicated. For simplicity, the cost calculated in (**A**,**C**,**E** left) is rearranged to appear decreasing as in (**B**,**D**,**F** left). Signature contribution is estimated upon fixing the first six signatures called by the backward (**A**,**C**,**E** right) and the forward (**B**,**D**,**F** right) feature selection. The graph shows the average contribution calculated within each simulated dataset: from top to bottom, 15 samples bearing n = 500, 1000, 2500 and 5000 mutations, 25 samples bearing n = 500, 1000, 2500 and 5000 mutations, and 50 samples bearing n = 500, 1000, 2500 and 5000 mutations. Signatures for which the contribution has been estimated are listed at the bottom of the heatmap: signatures included in the simulated datasets are shown in black and erroneously called signatures are shown in grey.

In the first step, *CaMuS* is fitting a panel of reference signatures to the spectrum of somatic mutations using a stepwise parameter selection (forward or backward) (Fig. 1). To assist the user with parameter selection, *CaMuS* outputs a curve where the cost associated to adding (forward procedure) or removing (backward procedure) signatures is displayed. Typically, the cost drops fast in the beginning followed by a tail towards the end of the curve. Signatures selected beyond the elbow of the curve are not notably modifying the cost and should not be included in the model, as they would probably lead to overfitting (Fig. 2). Thus, in comparison to other computational tools that do not offer a clear solution of how to select parameters, *CaMuS* is less prone to call spurious signatures (Fig. S2A, S2B)^12,16^.

Mutational signature inference tools such as *deconstructSigs*^10^ and *Mutalisk*^17^ use forward selection. To assess which parameter selection procedure (forward or backward) performs best in *CaMuS*, we simulated three mutational profiles as previously described (“Methods”). Each of the three profiles vary by its relative contribution of six Cosmic v2 mutational signatures: 4 ''sparse'' and 2 ''noisy'' signatures (Fig. S3). Signature.1 has the highest contribution in Simulation A, Signature.1 and Signature.3 are predominant in Simulation B, and all six signatures have comparable contributions in Simulation C (Fig. 2 and Fig. S3). We found that backward selection correctly assigned Signature.1 as being the feature, which mostly influences the cost in Simulation A. In contrast, during forward selection, Signature.1 is picked only in 6 of the 12 datasets as being the most influential feature (Fig. 2A,B). Within Simulation B, Signature.1 and Signature.3 are confidently called by the backward selection as the signatures best explaining the model (12 out of 12 datasets). Again, forward selection performed worse because it repeatedly missed Signature.3 (Fig. 2C,D). Lastly, forward parameter selection performed also poorly in Simulation C, as it assigned incorrect signatures in 5 out of 12 simulated datasets, whereas spurious signatures were assigned in only 2 datasets during backward selection process (Fig. 2E,F).

In conclusion, backward selection was more stable in our simulation study. However, both feature selection modes are implemented in *CaMuS* and the choice is left as an option to the user.

### Identification of novel signatures from unexplained data

**Figure 3:**
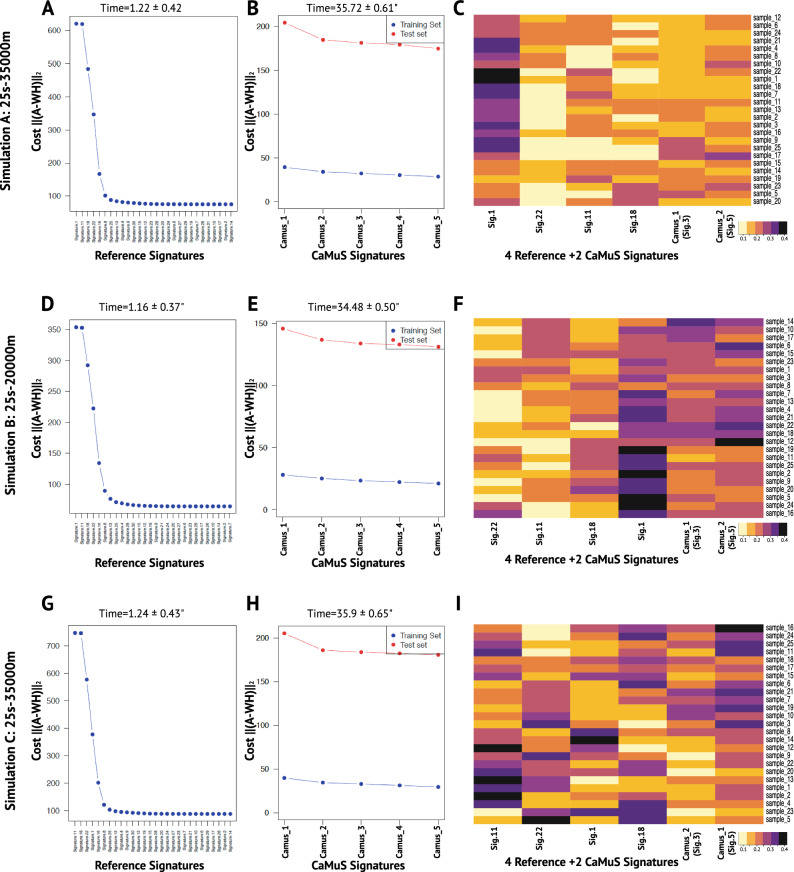
Performance of *CaMuS* on simulated data. (**A**–**C**) Simulation A, 25 samples and 35,000 mutations. (**D**–**F**) Simulation B, 25 samples and 20,000 mutations. (**G**–**I**) Simulation C, 25 samples and 35,000 mutations. (**A**,**D**,**G**) represent the cost associated to fit the reference signatures to the simulated datasets using the backward selection procedure. (**B**,**E**,**H**) represent the cost associated to inferring de novo signatures from the SNVs left unexplained upon fixing 4 reference signatures. The elbow of the test set indicates that adding signatures further does not improve model accuracy. (**C**,**F**,**I**) are heatmaps of the signature emissions. In (**C**), (**F**), and (**I**) the similarity of *CaMuS* signatures to the reference panel is estimated by selecting the smallest Hellinger Distance Score (HDS). In (**C**), Camus_1 is similar to Sig.3 (HDS = 0.31) and Camus_2 is like Sig.5 (HDS = 0.20). In (**F**), Camus_1 is similar to Sig.3 (HDS = 0.27) and Camus_2 is comparable with Sig.5 (HDS = 0.21). In (**I**), Camus_1 is like Sig.5 (HDS = 0.17) and Camus_2 is similar to Sig.3 (HDS = 0.26). The run time of each process is indicated on top of (**A**,**B**,**D**,**E**,**G**,**H**).

Signature fitting has the benefit to estimate the contribution of the reference signatures in a small dataset. However, in presence of previously undetermined mutational processes this may lead to spurious results. To circumvent this problem, *CaMuS* allows the user to evaluate whether additional de novo signatures can be extracted from mutation counts that are left unexplained during the fitting procedure (Fig. 1). Cross-validation is used to determine the number of de novo signatures that have to be added to the model. To demonstrate this functionality of *CaMuS* we used simulated data and prepared a reference panel signature based on Cosmic v2 but lacking Signature.3 and Signature.5 (Cosmic v2m). To this end, we initially fitted the Cosmic v2m to the simulated data (Fig. 3A,D,G). This step returned four simulated reference signatures accounting for most of the mutations in our datasets (i.e., Signature.1, Signature.11, Signature.18, and Signature.22, Fig. 3A,D,G). Next we evaluated the cost of adding de novo signatures (Fig. 3B,E,H). We found that across all simulated profiles, *CaMuS* could robustly predict and infere the two remaining signatures by the de novo inference procedure (see the elbow of the line in Fig. 3B,E,H and signature emissions in Fig. 3C,F,I and Fig. S4).

To assess minimal sample size to robustly infer mutational signatures, we tested simulated datasets with a varying number of mutations. To this end, we used the signature profile of Simulation A with a mutational burden from 2500 to 875,000 mutations. We found that with 2500–25,000 mutations it is possible to infer one de novo signature, while fitting up to four signatures. Increasing the size of the dataset to 62,500–125,000 mutations, *CaMuS* could robustly infer up to five de novo signatures, depending on how many reference signatures would be initially fitted to the data (Fig. S5A). Importantly, the prediction of novel parameters through the cost curve improved with sample size as well. Thus, we found that 875,000 mutations were necessary to confidently predict the presence of four fitted and two de novo signatures (Fig. S5, S6).

We next evaluated the computational performance of *CaMuS* (“Methods”). The run time required to perform the linear fitting procedure ranged from 0.95 to 1 s, while it took 33.40 to 35.10 s to evaluate the cost of adding de novo signatures (Fig. 3). Running the complete procedure took 0.06 GB of memory. Therefore, *CaMuS* is a computationally efficient tool to simultaneously fit and infere de novo mutational signatures.

### Comparison of *CaMuS* with *sigfit*

We benchmarked *CaMuS* against *sigfit*, a recently published method that, similarly to our tool, is able to simultaneously fit and extract novel signatures^12,13^. To this end, we used our previously simulated datasets (Fig. 3). While *sigfit* provides both NMF-equivalent and alternative Bayesian probabilistic model, we only assessed the performance of the NMF extraction method to have a direct comparison with *CaMuS*.

**Figure 4:**
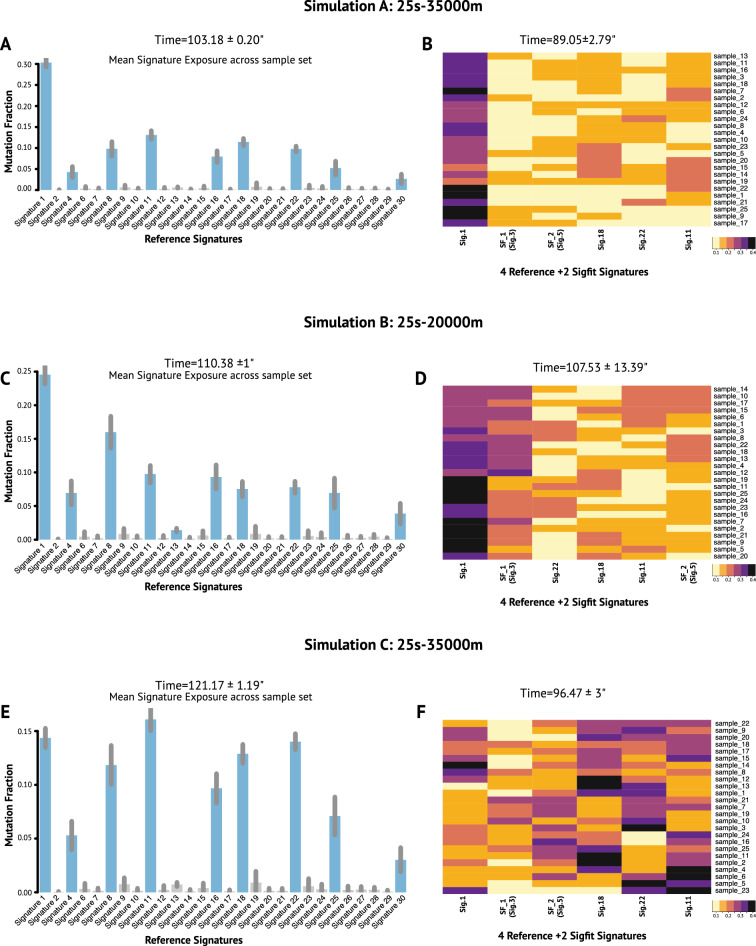
Performance of *sigfit* on simulated data. (**A**,**B**) Simulation A, 25 samples and 35,000 mutations. (**C**,**D**) Simulation B, 25 samples and 20,000 mutations. (**E**,**F**) Simulation C, 25 samples and 35,000 mutations. (**A**,**C**,**E**) Bar graphs showing the mean signature emissions of the reference signatures upon fitting. Error bars indicate 95% highest posterior density intervals. Grey bars indicate non-significant signature exposures. (**B**,**D**,**F**) are heatmaps of the signatures emissions: four reference signatures and two extracted de novo, SF_1 and SF_2. In (**B**), (**D**) and (**F**) the similarity of *sigfit* signatures to the reference panel is estimated by selecting the smallest Hellinger Distance Score. In (**B**), SF-1 is similar to Sig.3 (HDS = 0.14) and SF_2 is like Sig.5 (HDS = 0.15). In (**D**), SF_1 is like Sig.3 (HDS = 0.12) and SF_2 is similar to Sig.5 (HDS = 0.15). In (**F**), SF_1 is like Sig.3 (HDS = 0.13) and SF_2 is similar to Sig.5 (HDS = 0.13). The run time of each process is indicated on top of each graph.

First, we fitted the Cosmic v2m reference signatures to our simulated data. Unlike *CaMuS*, the fitting procedure with *sigfit* resulted in the prediction of 9 significantly active signatures, including the four present in our simulated dataset (Fig. 4A,C,E). Next, having fixed these four signatures, *sigfit* suggested up to four de novo signatures to be added to the model (Fig. S8). Thus, in a real case scenario, it is likely that *sigfit* may potentially lead to signature overfitting.

Interestingly, when we let *sigft* infere only two mutational signatures, the Hellinger distance score (HDS) indicates that *sigfit* is able to reconstruct Signature.3 and Signature.5 with slightly higher precision than *CaMuS*, with an HDS ranging from 0.12–0.15 and 0.31–0.17, respectively. However, the cost of the full model was consistently smaller when four fitted signatures and two de novo ones were inferred with *CaMuS* (Fig. S7). We next evaluated the computational performance of *sigfit*. The time to perform the fitting procedure in *sigfit* ranged from 103.18 to 121.17 seconds, and is therefore considerably longer than *CaMuS* (Fig. 3A,D,G). Similarly, the inference procedure used considerably higher amount of time (from 89.41 to 106.89 s on average) and of computer memory (0.58 GB) than *CaMuS* (Figs. 3B,E,H, 4B,D,F ). Thus, by applying the NMF method on synthetic data, we showed that the performance of *CaMuS* and *sigfit* is in a comparable range. However, we found two main advantages of *CaMuS* over *sigfit*: (i) *CaMuS* computational performance is superior to that of *sigfit*, as it has a shorter run time and uses considerably less computer memory (Figs. 3, 4, Table S1), (ii) *CaMuS* outputs a cost curve assisting the user with parameter selection to avoid overfitting. Parameter selection is not as straightforward in *sigfit* (Fig. S8).

### Application of *CaMuS* to whole genome sequencing data of neuroblastoma

Neuroblastoma is a paediatric tumour of the sympathetic nervous system. Its clinical course ranges from spontaneous regression to aggressive progression and metastasis^18^. We recently showed that mechanisms of telomere maintenance are a key feature of neuroblastoma tumours with poor prognosis^19,20^. Next, we wanted to study whether mutational signatures associated with the divergent clinical phenotypes were also in place. Thus, we applied *CaMuS* to our pre-treatment cohort of 56 genomes representing all clinical subtypes^20^. Signature fitting was performed using the novel reference signature panel recently published by the PCAWG consortium^6^. Backward parameter selection identified the following SNV-based signatures: Signature.18 and Signature.40 (Fig. S9A). Signature.18 has been previously identified in neuroblastoma, breast and stomach carcinomas datasets^4,21^. Recently, the aetiology of Signature.18 has been associated to DNA damage caused by reactive oxygen species (ROS) and a defective base excision repair. In fact, Signature.18 has been consistently reported in tumours carrying a germline bi-allelic mutation in the MUTYH gene, encoding a DNA glycosylase involved in the repair of oxidative DNA damage^21,22^. Signature.40 has been reported in many tumour types: it correlates with patient age in some tumours and it is similar to the age-related Signature.5 (HDS = 0.23)^6^. However, the exact aetiology of this signature is yet to be elucidated^6^. Cross-validation was next performed by fixing Signature.18 and Signature.40 and revealed that three de novo signatures (referred to as: Camus_1, Camus_2, and Camus_3) could be added (Fig. S9C). Camus_1 and Camus_2 mutation profiles resembled Signature.18 and Signature.5 with an HDS of 0.29 and 0.2, respectively (Fig. S9F, S9G). Camus_3 mutational profile was very similar to Signature.43 (HDS = 0.2), which has been previously described as a potential sequencing artefact^6^ and is therefore not included in the initial reference signatures panel^6^ (Fig. S9H). These observations led us to conclude that no substantially novel signatures could be discovered in our neuroblastoma dataset. However, the application of *CaMuS* highlighted the presence of mutational components that are private to our dataset and not present in the reference panel (i.e., Camus_1 and Camus_2). Furthermore, the inference of Camus_3 offered an example in real data of inferring signatures that are not included in the reference panel by our algorithm.

**Figure 5:**
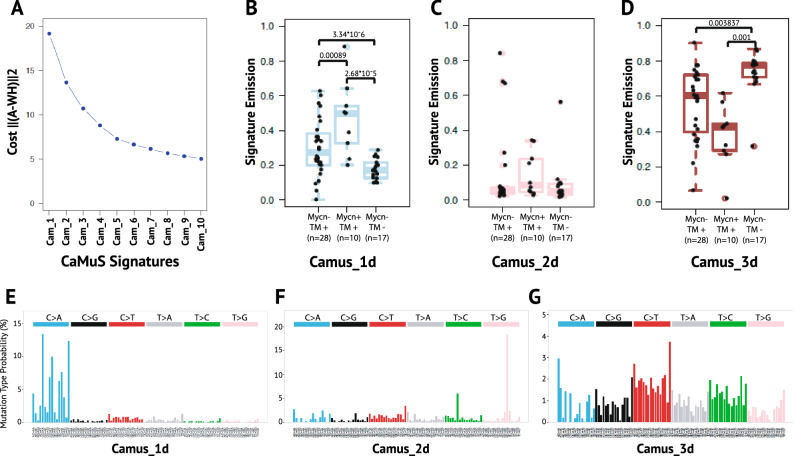
Mutational signatures in neuroblastoma. (**A**) The cost of inferring de novo signatures. (**B**–**D**) Boxplot of signatures emissions and their relative associations to neuroblastoma’s genotypes. *Mycn−* non *MYCN* amplified, *Mycn+ MYCN* amplified cases, *TM− *lack of an active telomere maintenance mechanism, *TM+* presence of an active telomere maintenance mechanism. Note that, Mycn+/TM− is not present because *MYCN* amplified neuroblastomas are always TM+^19,20^. The number of samples is indicated by *n*. Total number of samples sums to 55 instead of 56 because the genotypic information is missing for one cancer case. All p-values, indicated on top of the boxplots, are calculated from pairwise comparison between groups with correction for multiple hypothesis testing (i.e., pairwise wilcoxon rank sum tests). (**E**–**G**) De novo mutational signatures extracted by *CaMuS*.

In light of our previous observations, we decided to directly apply the NMF algorithm to the neuroblastoma genomes. This resulted in three mutational signatures (Fig. 5A): Camus_1d (Signature.18: HDS = 0.18, Fig. 5B,E), Camus_2d (Signature.43: HDS = 0.2, Fig. 5C,F) and Camus_3d (Signature.5: HDS = 0.2, Fig. 5D,G). Three de novo signatures similar to Camus_1d-Camus_3d were also detected with the R package *SomaticSignatures*^23^ (Fig. S2C, S2D). Unexpectedly, Camus_3d is not correlating with patient age using robust linear regression (*p-value* = 0.9539). Camus_1d, on the other hand, is strongly associated to patient cases with active telomere maintenance mechanisms (Fig. 5B–D). Furthermore, its contribution is effectively increased in those tumours also carrying the amplification of the *MYCN* proto-oncogene (Fig. 5B). Since Camus_1d is similar to Signature.18 of the reference signature panel, we searched for germline mutations within the *MUTYH* gene as previously suggested^22^. However, we could not find any germline mutation in the *MUTYH* coding region in our neuroblastoma dataset (Table S2). Therefore, our findings pose an interesting question if there is interplay between *MYCN* over-expression, ROS accumulation and active telomere maintenance mechanisms.

## Conclusion

*CaMuS* is a novel tool designed to enable the study of mutational signatures in a small cohorts of poorly characterised tumour types. Unlike most of the other tools, it offers the functionality to simultaneously fit reference signatures and extract novel signatures from the spectrum of somatic mutations present in cancer genomes. Having been implemented in C++, it only requires a minimal knowledge of the usage of the command line. *CaMuS* can be used to extract SNVs, as well as IDs and DBs mutational signatures (Fig. S10). We made use of simulated data to show that it can achieve stable results with relatively few samples and a few thousands mutations. When compared to *sigfit*, an R package with similar inference functionalities, *CaMuS* performs similar operations in a considerably lower amount of time and with considerably less memory usage. Furthermore, the use of the cost curve as a mean for parameter selection makes *CaMuS* more stable against signature overfitting. Finally, using neuroblastoma as an example, we showed the challenges that can be encountered during the interpretation of real data. Thus, more flexible tools, such as ours, represent a valuable resource to parsimoniously decipher the underlying mutational patterns.

## Supplementary information

Supplementary Information 1.

Supplementary Information 2.

Supplementary Information 3.

## Data Availability

Simulated data can be found in the Supplementary materials. The whole genome neuroblastoma sequencing data have been deposited at the European Genome-phenome Archive (https://www.ebi.ac.uk/ega/) under the accession number EGAS00001003244.
