# Supplementary material for: CaMuS: simultaneous fitting and de novo imputation of cancer mutational signature
Source: Sci Rep. 2020 Nov 9;10:19316. doi: 10.1038/s41598-020-75753-8 (PMC7653908; doi:10.1038/s41598-020-75753-8)
Supplement: Supplementary file 1 — Supplementary Information 1. [file 41598_2020_75753_MOESM1_ESM.pdf]

CaMuS: simultaneous fitting and de novo imputation of cancer mutational signature

Cartolano Maria, Abedpour Nima, Achter Viktor, Yang Tsun-Po, Ackermann Sandra, Fischer Matthias, and Peifer Martin*

Fig.S1

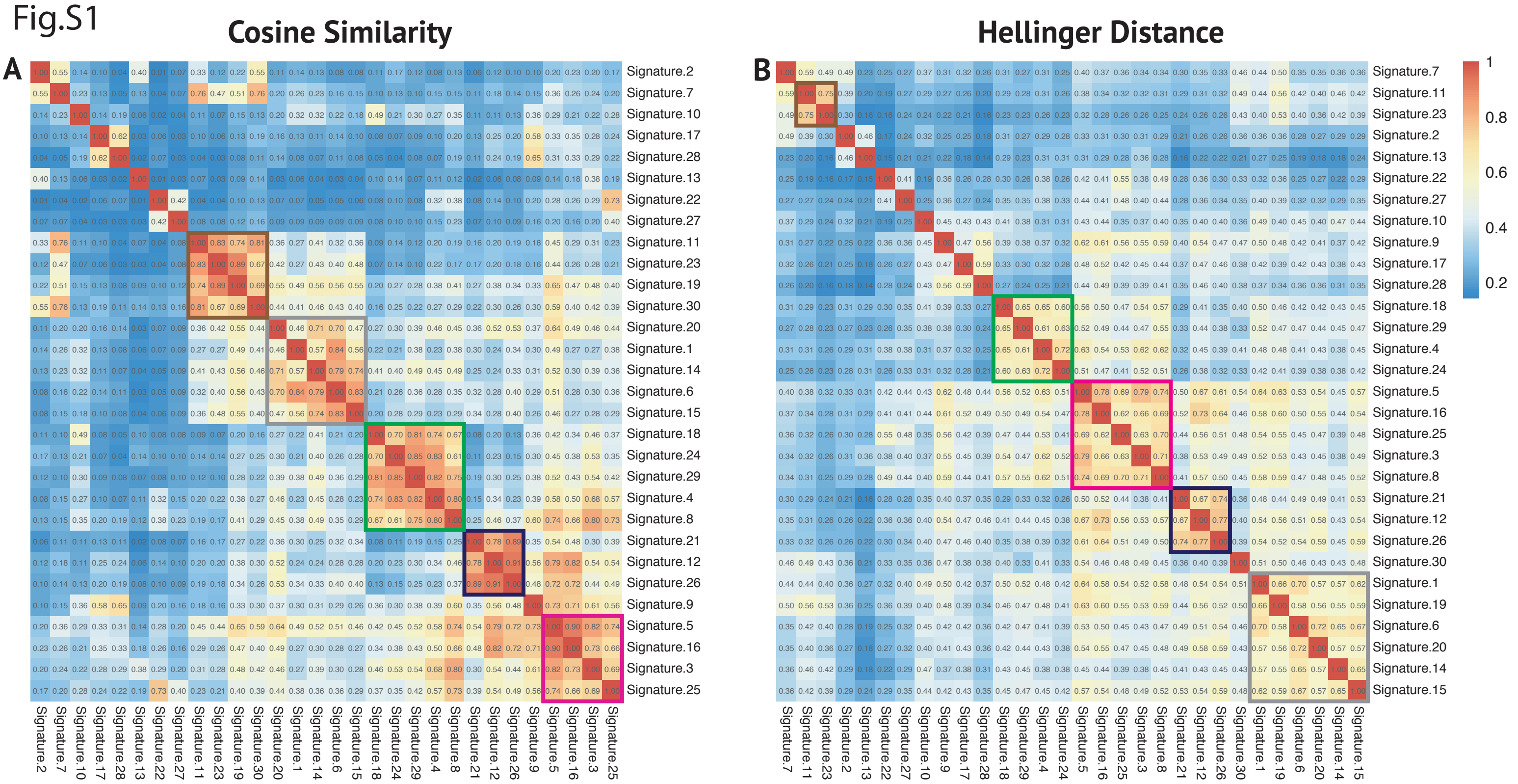

A
Cosine Similarity
Signature.2
Signature.7
Signature.10
Signature.17
Signature.28
Signature.13
Signature.22
Signature.27
Signature.11
Signature.23
Signature.19
Signature.30
Signature.20
Signature.1
Signature.14
Signature.6
Signature.15
Signature.18
Signature.24
Signature.29
Signature.4
Signature.8
Signature.21
Signature.12
Signature.26
Signature.9
Signature.5
Signature.16
Signature.3
Signature.25
B
Hellinger Distance
Signature.7
Signature.11
Signature.23
Signature.2
Signature.13
Signature.22
Signature.27
Signature.10
Signature.9
Signature.17
Signature.28
Signature.18
Signature.29
Signature.4
Signature.24
Signature.5
Signature.16
Signature.25
Signature.3
Signature.8
Signature.21
Signature.12
Signature.26
Signature.30
Signature.1
Signature.19
Signature.6
Signature.20
Signature.14
Signature.15
1
0.8
0.6
0.4
0.2

Fig. S2

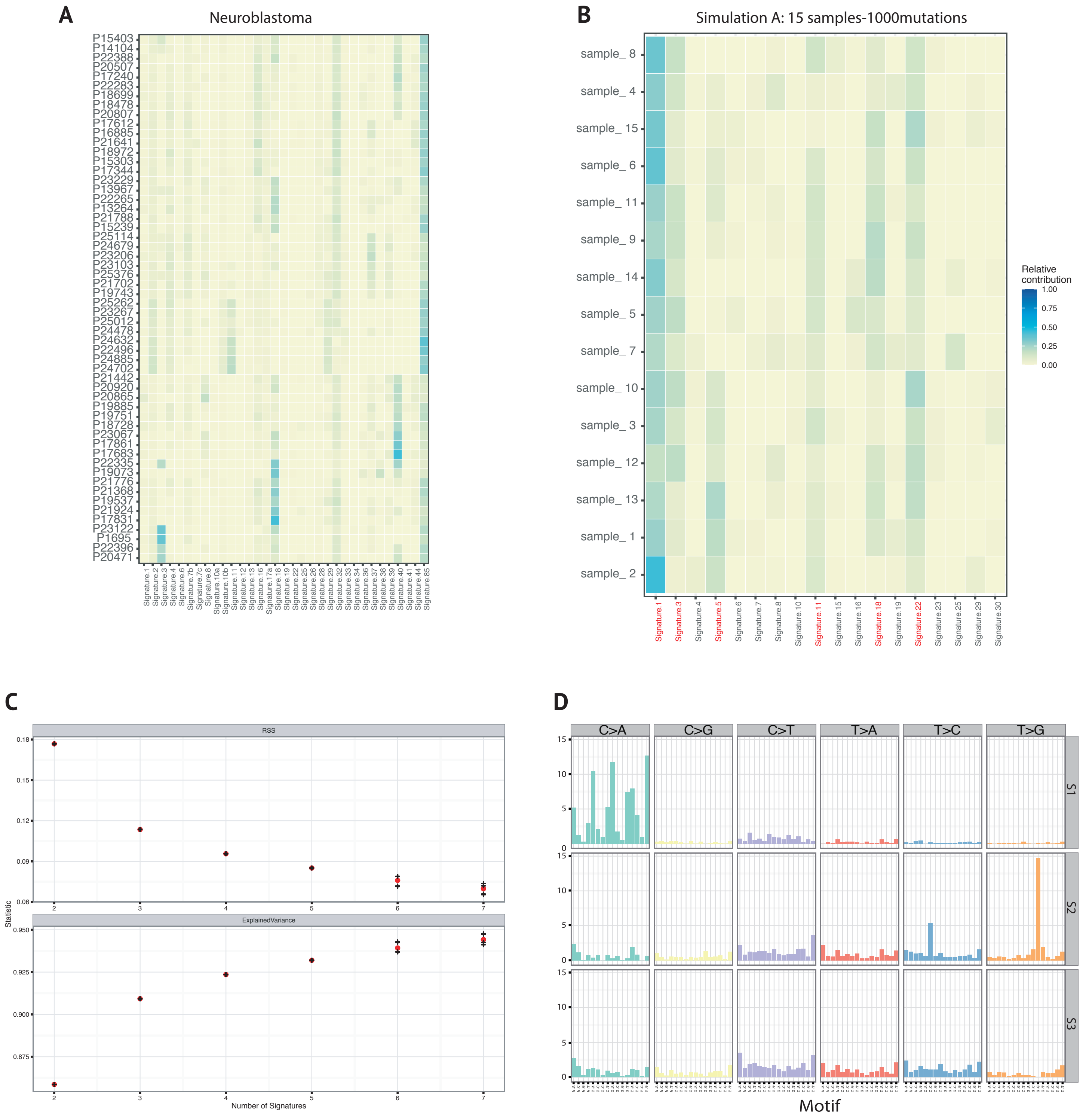

A
Neuroblastoma
B
Simulation A: 15 samples-1000mutations
sample_ 8
sample_ 4
sample_ 15
sample_ 6
sample_ 11
sample_ 9
sample_ 14
sample_ 5
sample_ 7
sample_ 10
sample_ 3
sample_ 12
sample_ 13
sample_ 1
sample_ 2
Signature.1
Signature.3
Signature.4
Signature.5
Signature.6
Signature.7
Signature.8
Signature.10
Signature.11
Signature.15
Signature.16
Signature.18
Signature.19
Signature.22
Signature.23
Signature.25
Signature.29
Signature.30
Relative contribution
1.00
0.75
0.50
0.25
0.00
C
RSS
ExplainedVariance
Statistic
Number of Signatures
D
C>A
C>G
C>T
T>A
T>C
T>G
S1
S2
S3
Motif

Fig. S3

A

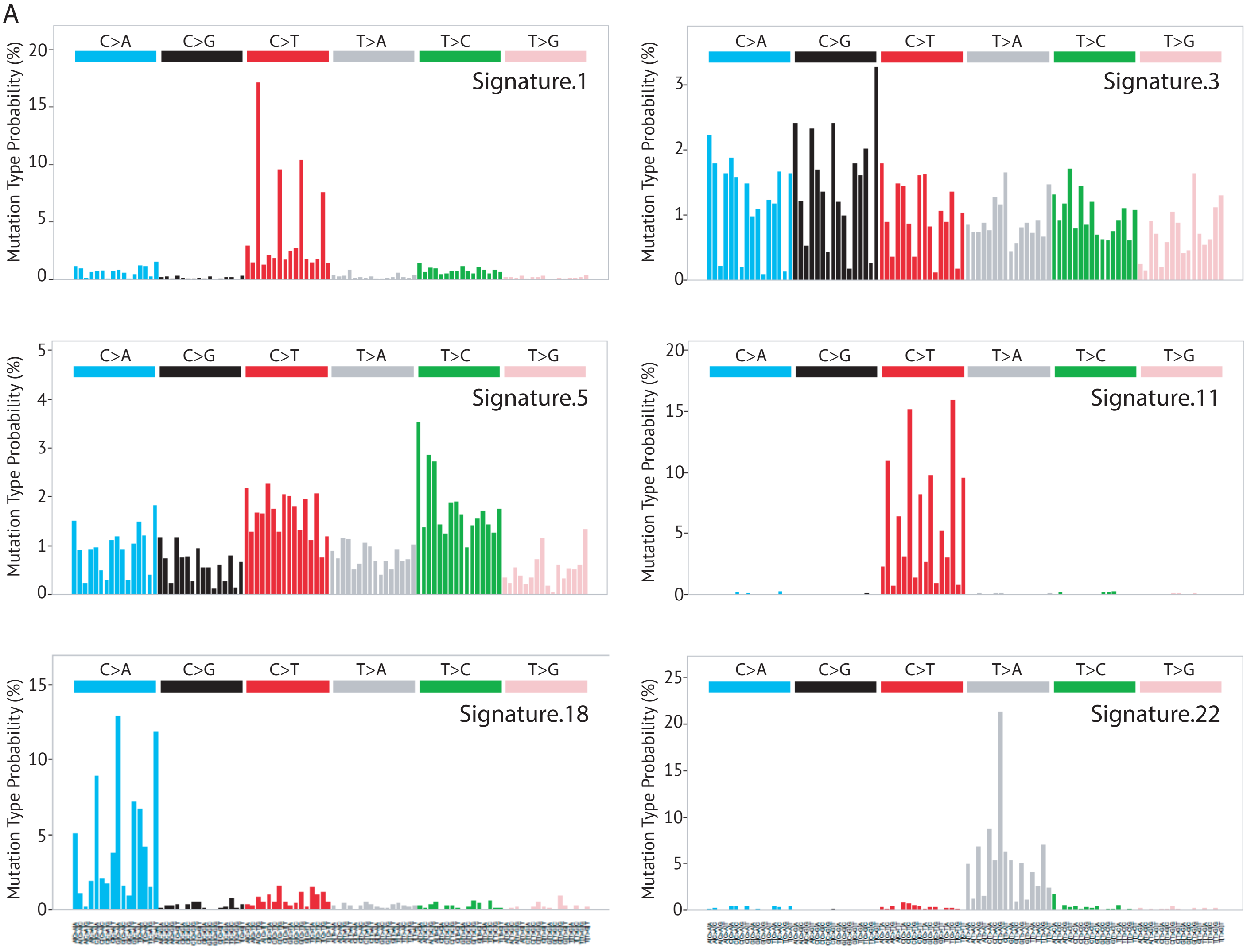

B

	Sig.1	Sig.3	Sig.5	Sig.11	Sig.18	Sig.22
Simulation A	0.5	0.3-0.1	0.3-0.1	0.3-0.1	0.3-0.1	0.3-0.1
Simulation B	0.5	0.5	0.3-0.1	0.3-0.1	0.3-0.1	0.3-0.1
Simulation C	0.3-0.1	0.3-0.1	0.3-0.1	0.3-0.1	0.3-0.1	0.3-0.1

Fig. S4

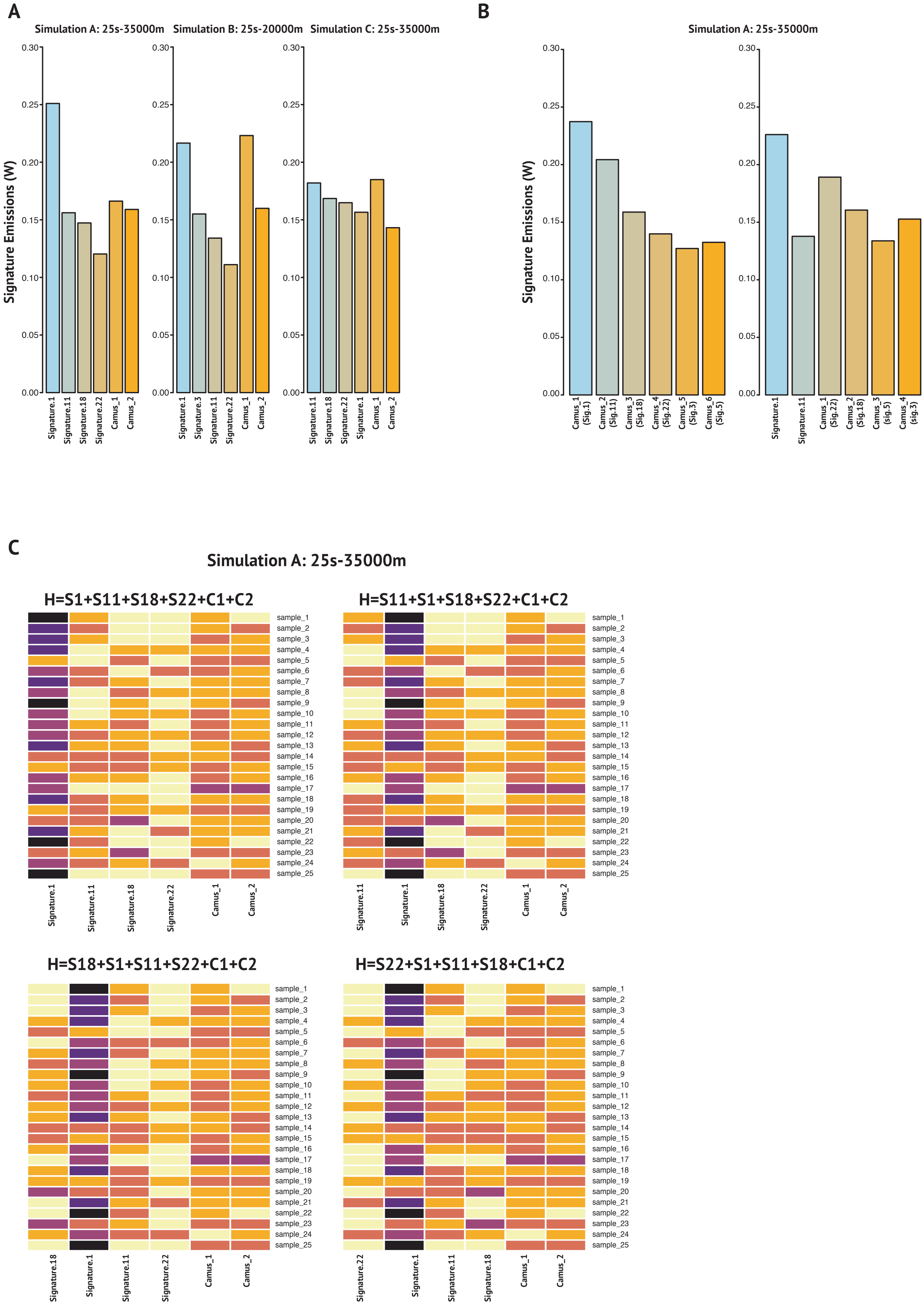
A
Simulation A: 25s-35000m
Simulation B: 25s-20000m
Simulation C: 25s-35000m
Signature Emissions (W)
Signature.1
Signature.11
Signature.18
Signature.22
Camus_1
Camus_2
Signature.3
B
Simulation A: 25s-35000m
Camus_1 (Sig.1)
Camus_2 (Sig.11)
Camus_3 (Sig.18)
Camus_4 (Sig.22)
Camus_5 (Sig.3)
Camus_6 (Sig.5)
Camus_1 (Sig.22)
Camus_2 (Sig.18)
Camus_3 (sig.5)
Camus_4 (sig.3)
C
Simulation A: 25s-35000m
H=S1+S11+S18+S22+C1+C2
H=S11+S1+S18+S22+C1+C2
H=S18+S1+S11+S22+C1+C2
H=S22+S1+S11+S18+C1+C2

Fig.S5

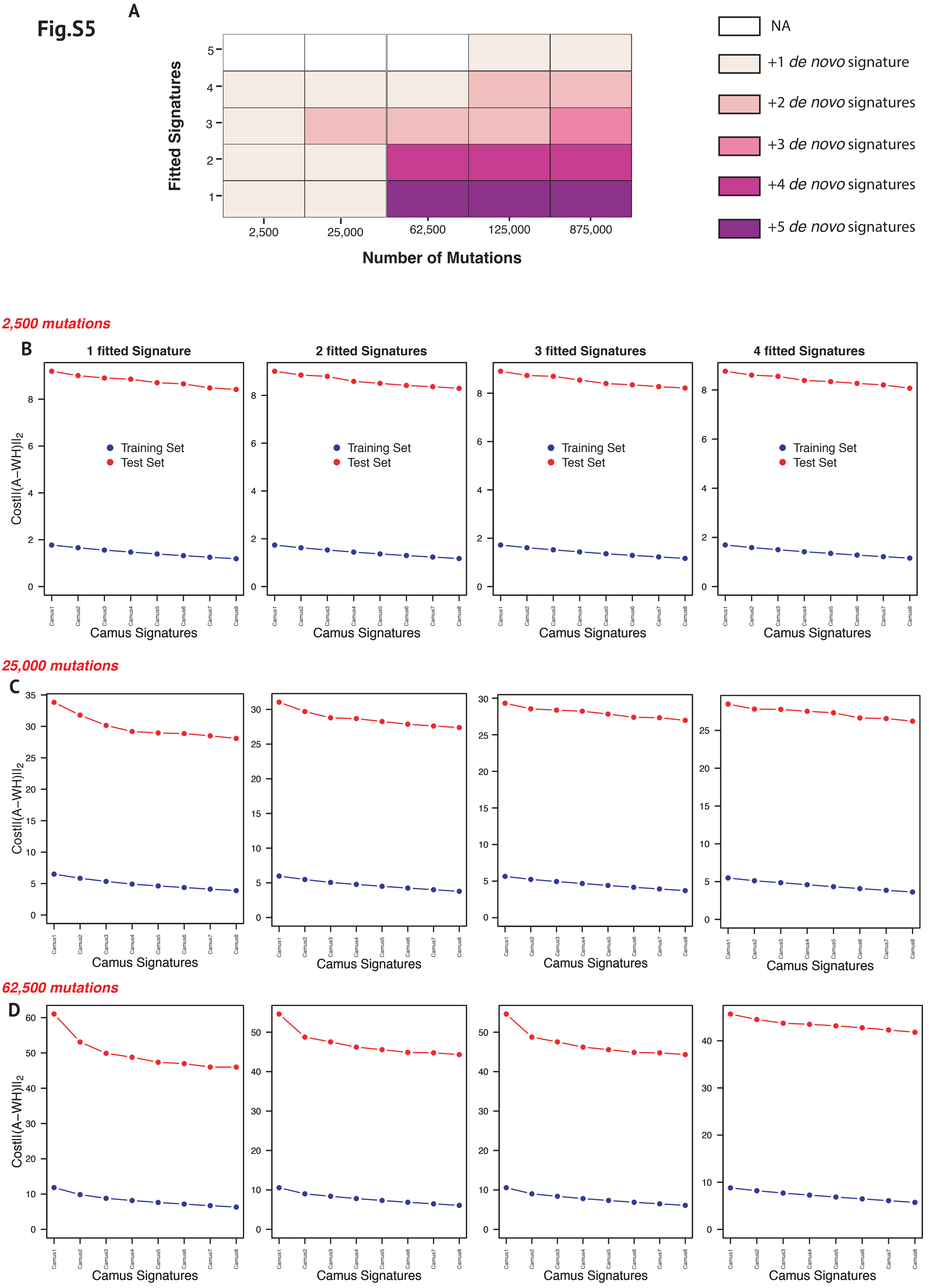
A
Fitted Signatures
5
4
3
2
1
2,500
25,000
62,500
125,000
875,000
Number of Mutations
NA
+1 de novo signature
+2 de novo signatures
+3 de novo signatures
+4 de novo signatures
+5 de novo signatures
2,500 mutations
B
1 fitted Signature
2 fitted Signatures
3 fitted Signatures
4 fitted Signatures
Training Set
Test Set
Cost||(A−WH)||2
Camus1
Camus2
Camus3
Camus4
Camus5
Camus6
Camus7
Camus8
Camus Signatures
25,000 mutations
C
62,500 mutations
D

Fig.S6

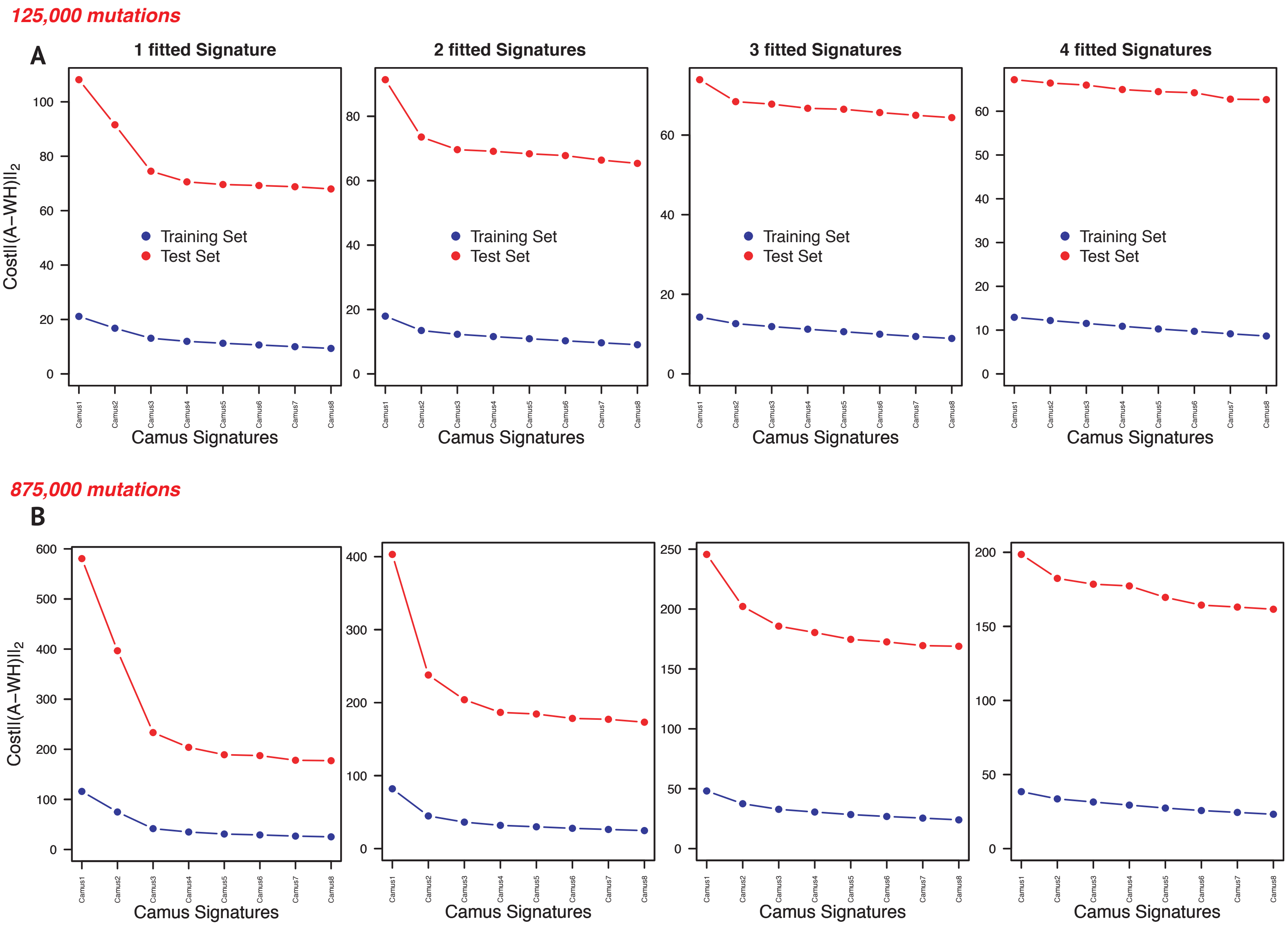

125,000 mutations
A
1 fitted Signature
2 fitted Signatures
3 fitted Signatures
4 fitted Signatures
Training Set
Test Set
Cost||(A−WH)||2
Camus Signatures
Camus1
Camus2
Camus3
Camus4
Camus5
Camus6
Camus7
Camus8
875,000 mutations
B

Fig.S7

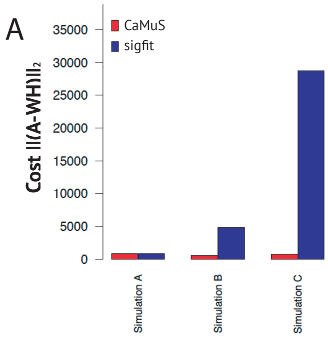
A
CaMuS
sigfit
Cost ||(A-WH)||2
35000
30000
25000
20000
15000
10000
5000
0
Simulation A
Simulation B
Simulation C

Fig.S8

A

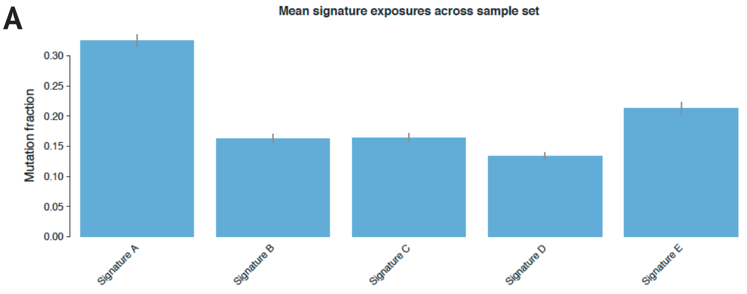

4 Reference +1 Sigfit Signatures

B

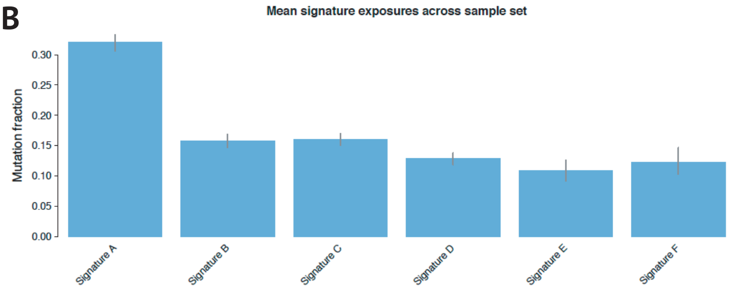

4 Reference +2 Sigfit Signatures

C

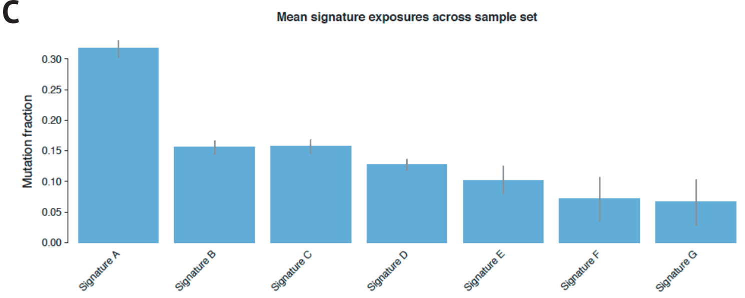

4 Reference +3 Sigfit Signatures

D

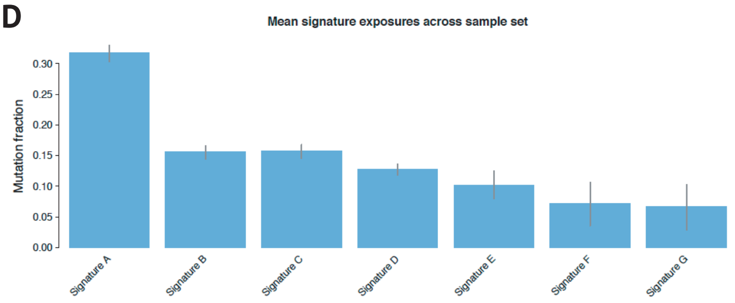

4 Reference +4 Sigfit Signatures

E

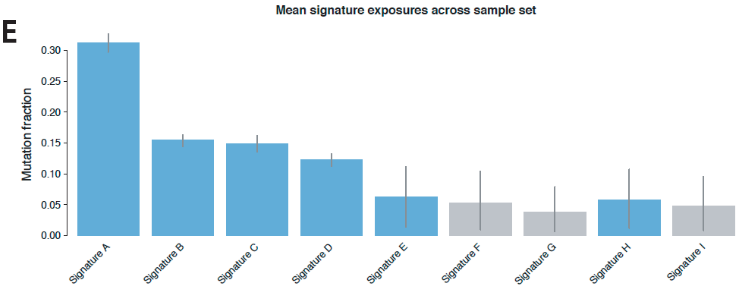

4 Reference +5 Sigfit Signatures

Fig. S9

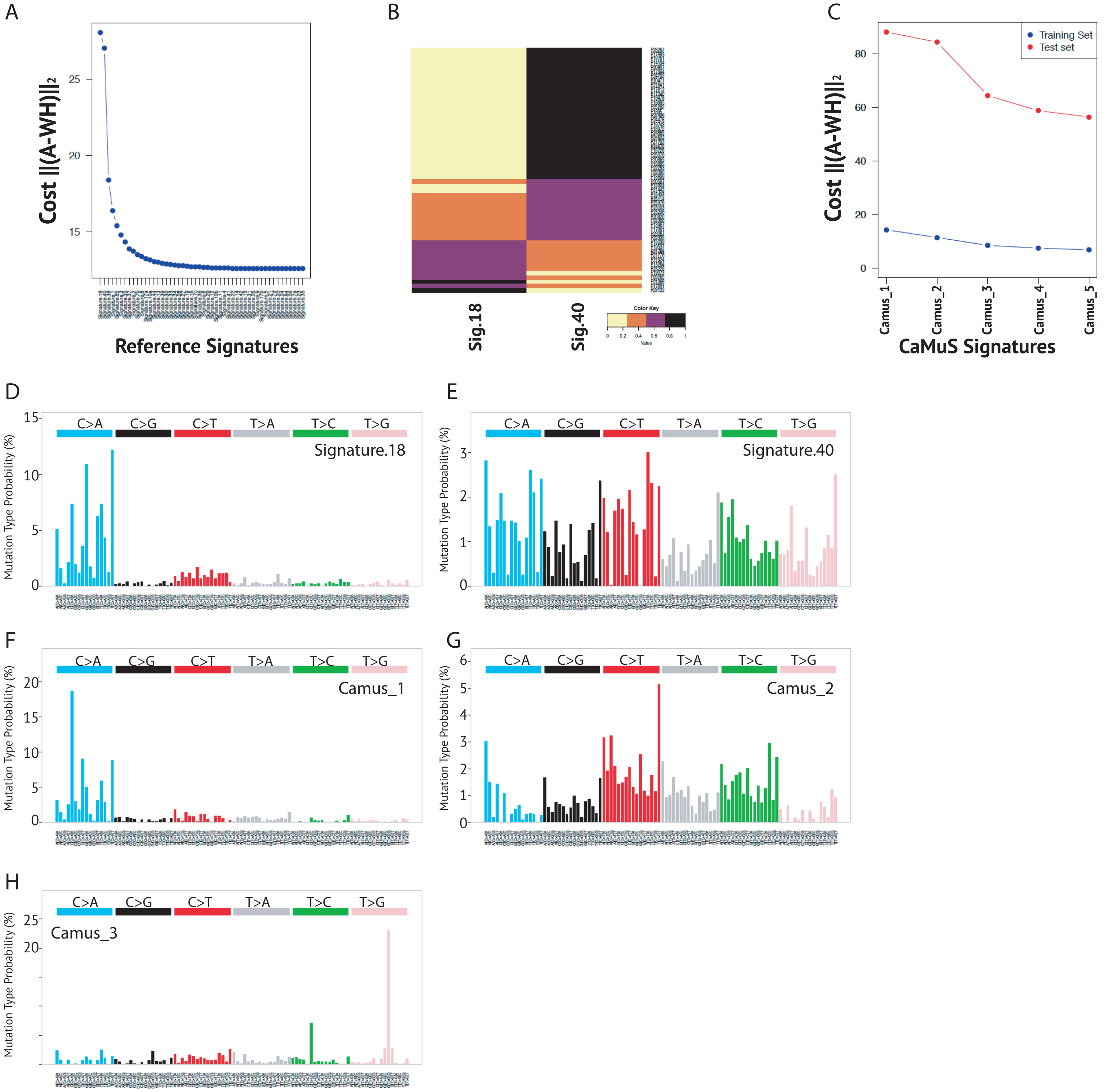
A
Cost ||(A-WH)||2
Reference Signatures
B
Sig.18
Sig.40
Color Key
Value
C
Training Set
Test set
Cost ||(A-WH)||2
Camus_1
Camus_2
Camus_3
Camus_4
Camus_5
CaMuS Signatures
D
Signature.18
Mutation Type Probability (%)
C>A
C>G
C>T
T>A
T>C
T>G
E
Signature.40
F
Camus_1
G
Camus_2
H
Camus_3

Fig. S10

A

Indel Signatures

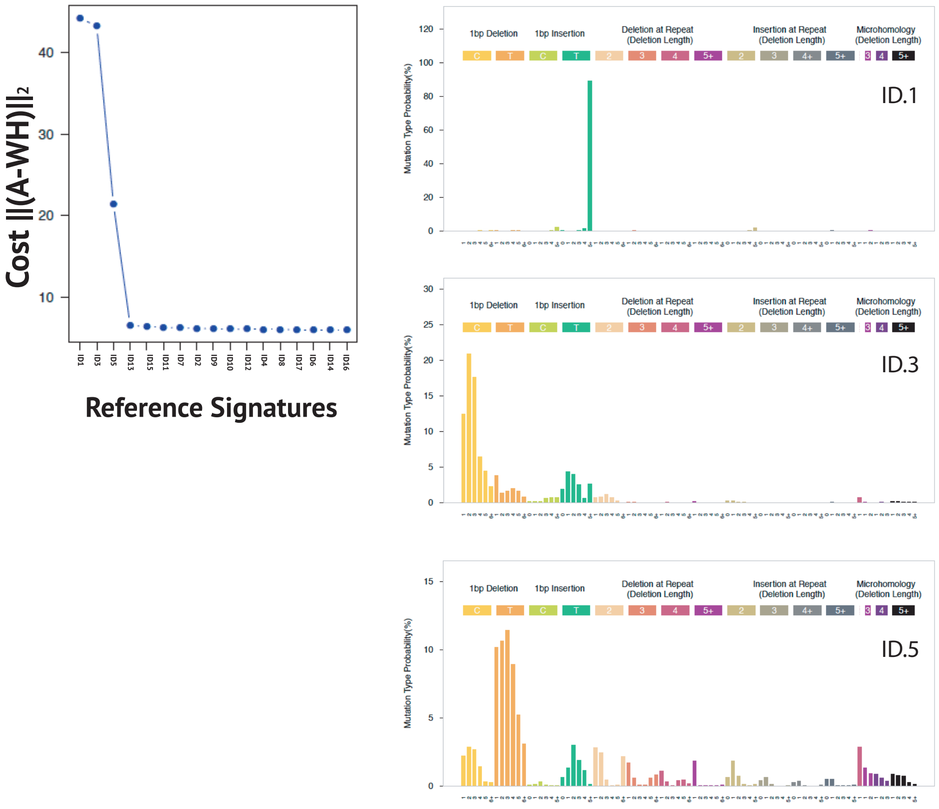

B

DBs Signatures

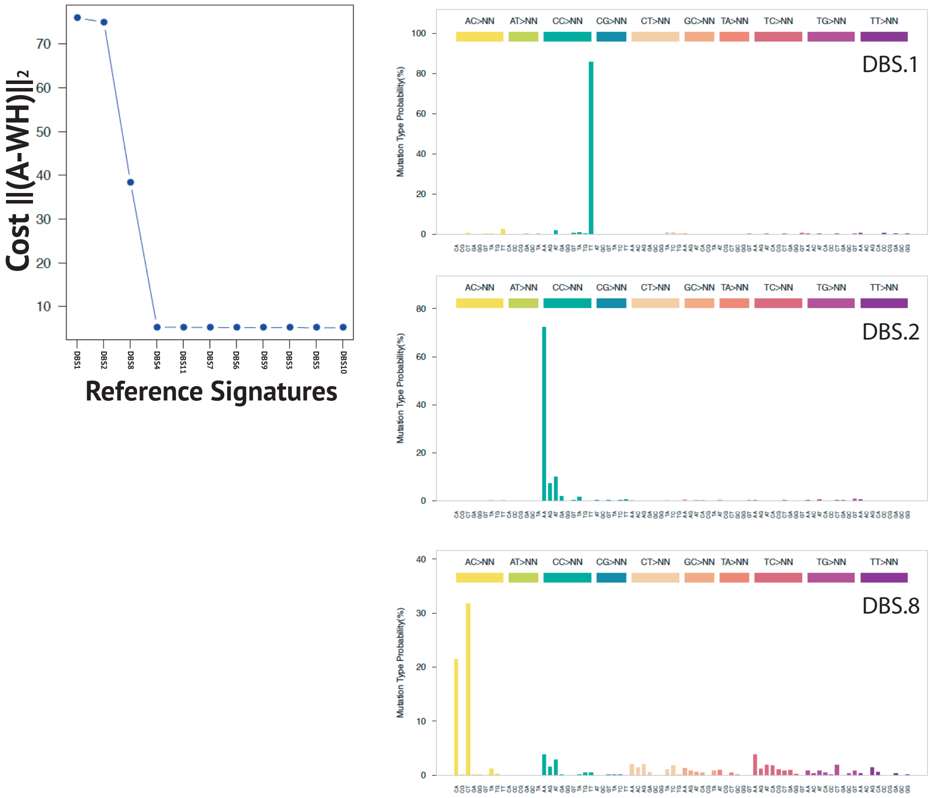

Table S1

Dataset	Procedure	CaMuS Walltime(s)	sigfit Walltime(s)	Speedup
Simulation A	Linear Fitting	0.95 ± 0.22	103.22 ± 0.19	5.37308229
Simulation A	Signature Extraction	34.90 ± 0.85	89.41 ±3.09	
Simulation B	Linear Fitting	1.00 ± 0.32	105.69 ±22.12	6.17963663
Simulation B	Signature Extraction	33.40 ± 0.60	106.89 ± 12.30	
Simulation C	Linear Fitting	0.95 ± 0.22	121.30 ± 1.14	6.05105409
Simulation C	Signature Extraction	35.10 ± 0.45	96.84 ± 3.29	
Memory Usage (GB)		*0.06*	*0.58*	
500_2500	Linear Fitting	13.3 ± 0.47	102.9 ± 0.72	39.38
500_2500	Signature Extraction	89.6 ± 0.75	3003.12 ± 46.97	
500_5000	Linear Fitting	13.75 ± 5.26	104.1 ± 5.37	41.37
500_5000	Signature Extraction	90.35 ± 1.46	3206.16 ±34.7	
500_35000	Linear Fitting	11.75 ±0.44	103.75 ± 0.64	171.4
500_35000	Signature Extraction	92 ±0.72	15613.03 ± 353.95	

Table S2

Gene Coordinate	Gene Feature	Tumor.ID
chr1:45795630	intron	NBL74
chr1:45795632	intron	NBL74
chr1:45795671	intron	NBL74
chr1:45795671	intron	NBL82
chr1:45795674	intron	NBL82
chr1:45795674	intron	NBL74
chr1:45795675	intron	NBL74
chr1:45795679	intron	NBL74
chr1:45795680	intron	NBL74
chr1:45795693	intron	NBL116
chr1:45795693	intron	NBL123
chr1:45795694	intron	NBL116
chr1:45795694	intron	NBL123
chr1:45795709	intron	NBL61
chr1:45795709	intron	NBL383
chr1:45795711	intron	NBL383
chr1:45795854	intron	NBL93
chr1:45795925	intron	NBL106
chr1:45795925	intron	NBL180
chr1:45795925	intron	NBL355
chr1:45795936	intron	NBL275
chr1:45796387	intron	NBL279
chr1:45796388	intron	NBL279
chr1:45796391	intron	NBL279
chr1:45796534	intron	NBL257
chr1:45797157	exon	NBL11
chr1:45800774	intron	NBL11
chr1:45802304	intron	NBL93
chr1:45802399	intron	NBL60
chr1:45803729	intron	NBL355
chr1:45803729	intron	NBL86
chr1:45803729	intron	NBL185
chr1:45804410	intron	NBL259
chr1:45804416	intron	NBL259
chr1:45804416	intron	NBL282
chr1:45804416	intron	NBL86
chr1:45804416	intron	NBL185
chr1:45804422	intron	NBL277
chr1:45804422	intron	NBL132

Table S2 (from page above)

Gene Coordinate	Gene Feature	Tumor.ID
chr1:45804425	intron	NBL11
chr1:45804429	intron	NBL93
chr1:45804430	intron	NBL93
chr1:45804430	intron	NBL74
chr1:45804430	intron	NBL151
chr1:45804430	intron	NBL347
chr1:45804430	intron	NBL81
chr1:45804430	intron	NBL61
chr1:45804435	intron	NBL185
chr1:45804435	intron	NBL383
chr1:45804435	intron	NBL81
chr1:45804609	intron	NBL86
chr1:45804856	intron	NBL60
chr1:45804900	intron	NBL275
chr1:45804900	intron	NBL389
chr1:45804901	intron	NBL389
chr1:45804907	intron	NBL275
chr1:45804907	intron	NBL339
chr1:45805562	intron	NBL54
chr1:45805562	intron	NBL275
chr1:45805562	intron	NBL257
chr1:45805569	intron	NBL257

References

1 R Core Team. R: A Language and Environment for Statistical Computing (R Foundation for Statistical Computing, Vienna, https://www.R-project.org).
