# Supplementary material for: CaMuS: simultaneous fitting and de novo imputation of cancer mutational signature
Source: Sci Rep. 2020 Nov 9;10:19316. doi: 10.1038/s41598-020-75753-8 (PMC7653908; doi:10.1038/s41598-020-75753-8)
Supplement: Supplementary file 3 — Supplementary Information 3. [file 41598_2020_75753_MOESM3_ESM.docx]

**Figure S1: Comparison between the Hellinger distance and the cosine similarity measures.** Heatmaps showing signature similarity between the Cosmic v2 mutational signatures. Similarity is estimated using the cosine similarity (A) and the Hellinger distance measures (B). Numbers inside the cells indicate the actual measures. Signature identity=1. Clusters of similar signatures are boxed to allow comparison between the two methods.

**Figure S2: *CaMuS*’ s performance in comparison to *SomaticSignatures* and *MutationalPatterns* R packages.** (A-B) The contribution of the Cosmic signatures v2 and v3 as estimated by *MutationalPatterns* in the neuroblastoma dataset (A) and in one of the simulated datasets (i.e. Simulation A, 15samples-1000mutations)(B). In both instances, signatures with an arbitrarily chosen contribution >100 are selected. The lack of a procedure for parameter selection, as the one offered by *CaMuS*, leads to overfitting. When applied to the neuroblastoma dataset, *MutationalPatterns* estimate signature.85 being present in almost all samples. Signature.85 is normally found in cancers of lymphoid origin and therefore it is likely to be in neuroblastoma a spurious call. Labels in red in panel B indicate the simulated signatures. Panels C-D shows *de novo* signature parameter selection and inference as estimated by the R package^24^ *SomaticSignatures* applied to the neuroblastoma dataset.

**Figure S3: Overview of the simulated datasets.** A) The mutational signatures according to Cosmic v2. B) Schematic diagram of the three simulation profiles and the contribution assigned to each signature defined as the probability of success in a multinomial distribution.

**Figure S4: Robustness of the signatures emissions estimated by *CaMuS*.** A) Barplots of mean signatures emissions upon 50 *CaMuS* runs using a random seed to initialize the algorithm. The simulated datasets are those used in Figure 3 and Figure 4. The estimated contributions were identical in each run (thus, no standard deviation can be displayed). B) Barplots of mean signature emissions with $H=$6 *de novo* signatures (left) and $H=$ 2 fitted signatures plus 4 *de novo* (right). Signature 1 is the most dominant signature in both examples. In Panel B, left, Camus_1 is Signature.1-like (HDS=0.28), Camus_2 is Signature.11-like (HDS=0.18), Camus_3 is Signature.18-like (HDS=0.22), Camus_4 is Signature.22-like (HDS=0.17), Camus_5 is Signature.3-like (HDS=0.23), Camus_6 is Signature.5-like (HDS=0.33). In Panel B, right, Camus_1 is Signature.22-like (HDS=0.26), Camus_2 is Signature.18-like (HDS=0.21), Camus_3 is Signature.5-like (HDS=0.30), Camus_4 is Signature.3-like (HDS=0.33). C) Four heatmaps of signatures emissions estimated dataset by changing the order of the signatures called by the backward selection within Simulation A.

**Figure S5: Optimal sample size to confidently detect novel signatures with *CaMuS*.**

A) Schematic diagram of the number of signatures that can be detected *de novo* upon a variable a number of fixed signatures. The x-axis represents datasets with increasing number of mutations and the y-axis is the number of signatures fitted. The datasets contain six simulated signatures following the mutational signature profile of Simulation A. B-D) cross-validation results upon fitting 1-4 signatures (left to right) in datasets with 2,500 mutations (B), 25,000 mutations (C), 62,500 mutations.

**Figure S6: Optimal sample size to confidently detect novel signatures with *CaMuS.*** The datasets contain six simulated signatures following the mutational signature profile of Simulation A. A-B) cross-validation results upon fitting 1-4 signatures (left to right) in datasets with 125,000 mutations (B), 875,000 mutations.

**Figure S7: The performance of *CaMuS* (red) and *sigfit* (blue) in reconstructing the original mutational catalogue.** Simulation A and Simulation C are simulated datasets of 25 samples and 35,000 mutations, while Simulation B is made of 25 samples and 20,000 mutations. The y-axis is the Frobenius norm where $H$ and $W$ are the signature and emission matrices generated by both tools once having fixed four reference signatures and extracted two *de novo* ones.

**Figure S8: Model parameter selection in *sigfit*.** A-E) Bar graphs of signature exposures with Signature A to Signature D corresponding to the Cosmic v2 Signature.1, Signature.11, Signature.18 and Signature.22. Signature E to Signature I correspond to the novel signatures inferred. The blue bars indicate the significantly active signatures estimated by the model. Thus, s*igfit* estimates 1 to 4 active novel signatures. On the contrary, the cost curve outputed by *CaMuS* and shown in Fig. 3B clearly suggests the addition of only two novel signatures.

**Figure S9: Application of *CaMuS* to neuroblastoma.** (A) The cost of adding the reference signatures to the neuroblastoma cohort. (B) Heatmap of the emission of the three reference signatures selected. (C) Cross-validation test of adding *de novo* features after having selected two reference signatures. (D-E) Signature.18 and Signature.40 mutation probabilities diagram. (F-H) Diagram of the mutational signatures extracted by *CaMuS* after cross-validation.

**Figure S10: Extraction of IDs and DBs signatures from simulated data.** A) IDs signatures ID.1, ID.3 and ID.5 extracted from a simulated dataset consisting of 15 samples and 1000 mutations. The cost curve is displayed on the left. The mutation probability diagrams generated by *CaMuS* are indicated on the right side. B) DBs signatures DB.1, DB,2 and DB.8 extracted from a simulated dataset consisting of 15 samples and 1000 mutations. The cost curve is displayed on the left. The mutation probability diagrams generated by *CaMuS* are indicated on the right side.

**Table S1**: *CaMuS* and *sigfit* run time.

**Table S2**: Germline mutations in the *MUTYH* gene observed in our WGS neuroblastoma cohort.
